# Arginase promotes immune evasion of *Echinococcus granulosus* in mice

**DOI:** 10.1186/s13071-020-3919-4

**Published:** 2020-02-06

**Authors:** Shengkui Cao, Wenci Gong, Xiaofan Zhang, Meng Xu, Ying Wang, Yuxin Xu, Jianping Cao, Yujuan Shen, Jiaxu Chen

**Affiliations:** 1 National Institute of Parasitic Diseases, Chinese Center for Disease Control and Prevention, Shanghai, 200025 China; 2 Chinese Center for Tropical Diseases Research, Shanghai, 200025 China; 3 WHO Collaborating Centre for Tropical Diseases, Shanghai, 200025 China; 4 National Center for International Research on Tropical Diseases, Ministry of Science and Technology, Shanghai, 200025 China; 5 Key Laboratory of Parasite and Vector Biology, Ministry of Health, Shanghai, 200025 China

**Keywords:** *Echinococcus granulosus*, Arginase, Immunosuppression, INOS, Peritoneum

## Abstract

**Background:**

Cystic echinococcosis is a chronic disease caused by infection with the larvae of *Echinococcus granulosus*. The parasite’s ability to establish persistent infection is partly due to its evolving immune evasion strategies. One strategy may involve the protective effect of arginase, which impedes the control of pathogens or tumors, whereas it remains largely unknown during *E. granulosus* infection. Here, we analyzed whether arginase was produced in peritoneal cells and assessed its role in immunosuppression in mice infected with protoscoleces of *E. granulosus*.

**Methods:**

BALB/c mice injected with protoscoleces of *E. granulosus* were used to evaluate the expression of arginase (ARG) in mRNA and protein levels. The profiles of ARG-1 expression in peritoneal cells and CD3*ζ* expression in T cells from spleens were assessed at different time points (3, 6, 9 and 12 months post-infection) by flow cytometry. *In vitro*, peritoneal cells were co-cultured with purified T cells in a transwell system, and the levels of CD3*ζ* re-expression were compared by flow cytometry. Meanwhile, the changes of l-arginine and its related metabolites in serum were tested.

**Results:**

Compared to the control group, the peritoneal cells from infected mice showed higher levels of ARG-1 mRNA and protein, unchanged ARG-2 and iNOS. Enhanced ARG-1 expression was present in SSC^low^CD11b^+^F4/80^+^, CD11b^+^CD11c^+^, CD11b^+^Gr-1^+^Ly-6C^+^Ly-6G^−^, CD11b^+^Gr-1^+^Ly-6C^−^Ly-6G^+^, CD11b^+^Gr-1^+^ and CD11b^+^Ly-6G^+^ cells. The proportion of cells and the proportion of ARG-1 expression in corresponding cells exhibited a rising trend along with the extension of infection time, except for fluctuations in SSC^low^CD11b^+^F4/80^+^ and CD11b^+^CD11c^+^ cells at 12 months post-infection, whereas the expression of CD3*ζ* chain in CD4^+^ and CD8^+^ T cells showed a descending trend. Purified T cells showed declined re-expression of CD3*ζ* when co-cultured with peritoneal cells from infected mice, and CD3*ζ* was regenerated by supplement of l-arginine or arginase inhibitor BEC, rather than NOS inhibitor l-NMMA or catalase. Meanwhile, the concentrations of l-arginine, l-citrulline and NO decreased, and those of l-ornithine and urea increased in serum post-infection.

**Conclusions:**

Our findings demonstrated that ARG-1 expression is enhanced in multiple myeloid cells from peritoneum and promotes immune evasion of *E. granulosus* in mice by inhibiting the expression of T cell receptor CD3*ζ* chain and antagonism against iNOS.
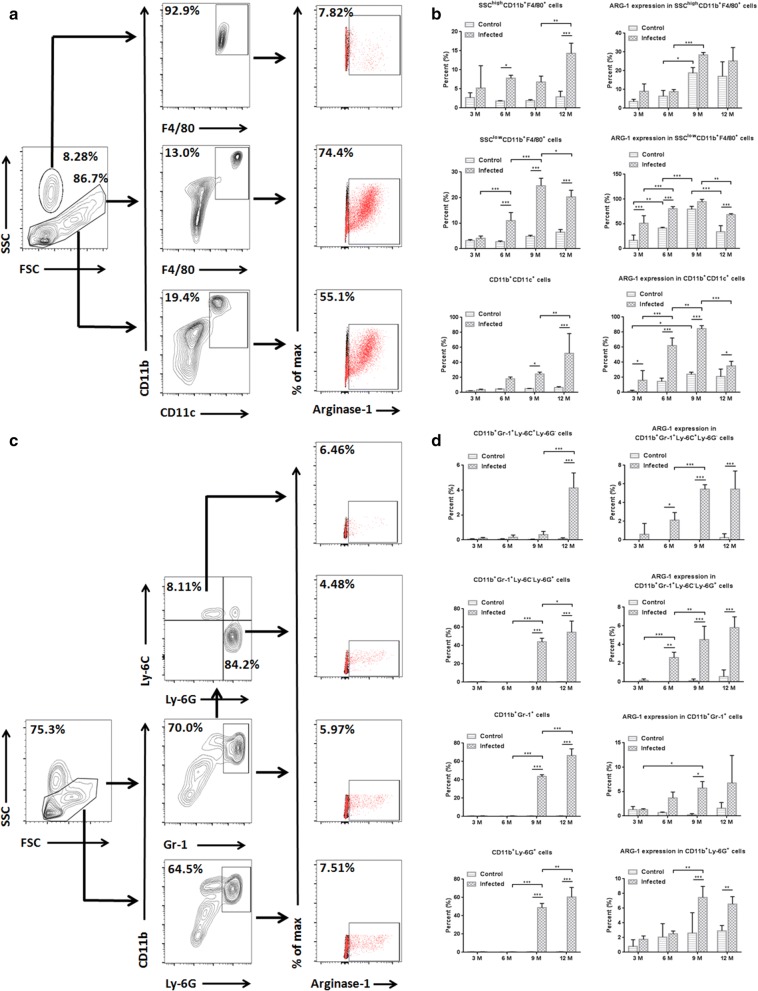

## Background

Arginase is a ubiquitous and manganese-containing enzyme, including two isoforms: arginase-1 (ARG-1), localized in the cytosol; and arginase-2 (ARG-2), located in the mitochondria. During the urea cycle, the final reaction (converting l-arginine to l-ornithine and urea) is catalyzed by arginase, eliminating toxic ammonia [1]. However, excessively elevated arginase caused by infections or cancers could be detrimental for the host [2]. This mainly has two pathologically negative effects: (i) l-arginine starvation *in vivo*; and (ii) antagonism against nitric oxide (NO) synthase (NOS) for the same substrate (catalyzing l-arginine to l-citrulline and NO). The former could result in decreased expression of the T cell receptor *ζ* chain (CD3*ζ*) which is the principal signal transduction element of the receptor and an impairment in T cell function [3, 4], serving as a survival strategy for bacteria and parasites [5]. The latter reduces NO production which is antibacterial and beneficial to anti-tumor immunity [6]. NOS has three isoforms: neuronal NOS (nNOS); inducible NOS (iNOS); and endothelial NOS (eNOS). Among these, nNOS and eNOS are expressed constitutively, prevalent in neuronal tissue and endothelial cells, respectively, to produce NO at a low to moderate rate. iNOS is regulated at the expression level, present in various immune cells, and produces NO at a high rate [7, 8]. iNOS-derived NO is needed for signal transduction and microbial defense. In addition to these immunoregulatory effects, ARG-1 expressed by myeloid cells has been involved in promoting tissue repair and wound healing because the l-ornithine is a precursor of proline and polyamines [9, 10]. However, in cutaneous leishmaniasis and some tumor models with high arginine-succinate synthetase (ASS1) activity which could produce l-arginine from citrulline, arginase seems to be dispensable for the long-term persistence to pathogens [11, 12]. Here, we asked whether arginase was produced and whether its immunosuppression effect was present in *Echinococcus granulosus-*infected mice, although other immune evasion mechanisms have been found in previous studies [13, 14].

*Echinococcus granulosus* belongs to a platyhelminth cestode, whose larval stage is called a hydatid cyst and is filled with hydatid cyst fluid and protoscoleces (Eg-PSC). These larvae grow within intermediate hosts and cause cystic echinococcosis, commonly prevalent in pastoral regions around the world [15]. Although specific immune responses are present, *E. granulosus* infection persists in the host over many years [16]. It is largely because that the parasite’s evasion strategies evolve to avoid being eliminated by the immune system.

Previously, we found accumulation of ARG-1 in monocytic myeloid-derived suppressor cells (M-MDSCs) from spleens [17] and Peng et al. [18] showed increased ARG-1 in macrophages from livers after *E. granulosus* infection. However, there is no report about the change of arginase in peritoneal cells *in vivo* and its immunosuppressive effect. Enterocoelia is one of the major pathogenic sites occupied with hydatid cysts, especially in the mouse model [15]. We analyzed the arginase expression profiles in multiple peritoneal myeloid cells and assessed its immunosuppression mechanism in the process of *E. granulosus* infection. Our results showed that elevated expression and activity of ARG-1, but not ARG-2, were present in multiple cells post-infection, along with declined expression of T cell receptor CD3*ζ* chain in CD4^+^ and CD8^+^ T cells. Furthermore, the re-expression of CD3*ζ* was inhibited by arginase *in vitro*, and ARG-1 antagonized iNOS *in vivo*. These observations revealed an immunoregulatory mechanism by which *E. granulosus* evades damage from the host.

## Methods

### Mice, parasites and modeling

Female BALB/c mice (aged 6–8 weeks) were purchased from SLAC Laboratory Animal Co. Ltd., Shanghai, China, under aseptic conditions. The Eg-PSC were obtained by puncturing the fertile hydatid cysts within livers of naturally infected sheep from Xinjiang Uygur Autonomous Region, China. The parasites were washed five times using a sterile 0.9% NaCl solution supplemented with 100 U/ml penicillin and 100 μg/ml streptomycin. The vitality of PSC from each individual liver was determined by the trypan blue dye exclusion method and those exhibiting over 90% vitality were used for infection.

Fifty BALB/c mice were intraperitoneally inoculated with a 200 μl sterile suspension containing 2000 live Eg-PSC in 0.9% NaCl, and fifty controls were inoculated with 200 μl 0.9% NaCl. All mice were maintained in specific pathogen-free conditions and fed with standard laboratory food and water.

Genotype identification of Eg-PSC was carried out according to Nakao et al. [19] using the primers (5′-TTG AAT TTG CCA CGT TTG AAT GC-3′ and 5′-GAA CCT AAC GAC ATA ACA TAA TGA-3′) targeting cytochrome *c* oxidase subunit 1 gene.

### Western blot analysis

Peritoneal cells were isolated immediately after the infected and control mice were sacrificed under sterile conditions 9 months post-infection. Macrophages were separated using a Macrophage Isolation Kit (Peritoneum) (Miltenyi Biotec, Bergisch Gladbach, Germany) and the purity (F4/80^+^) exceeded 90%, as determined by flow cytometry. Macrophages and non-macrophage cells were respectively lysed for 30 min on ice in a RIPA solution containing protease inhibitors. Lysates were then separated by 10% SDS-PAGE and transferred to a polyvinylidene difluoride membrane (Merck Millipore, Darmstadt, Germany). After blocking of non-specific binding sites, the respective blots were incubated with different primary antibodies [anti-Arginase-1, anti-Arginase-2, anti-iNOS and anti-β-actin (Cell Signaling Technology, Danvers, MA, USA)] and the respective HRP-conjugated secondary antibodies. The results were visualized using the ECL detection system (Merck Millipore) and recorded with a Universal Hood II Imager (Bio-Rad, California, USA). Band intensities were evaluated using image J (NIH, Bethesda, MD, USA).

### Immunofluorescent assay

Peritoneal cells were cultured in a Millicell EZ Slide (Merck Millipore) for 48 h. Cells which grew in the slide were fixed, rinsed and stained using anti-Arginase-1 antibody and anti-Arginase-2 antibody, respectively (Cell Signaling Technology). After washing with PBS, the slides were incubated with a secondary antibody conjugated with fluorescein (Life Technologies, Carlsbad, CA, USA). For visualizing nuclei, the slides were mounted with DAPI (Cell Signaling Technology). Images were captured with a fluorescence microscope (Leica DM IRB, Germany).

### Reverse transcription quantitative real-time PCR (RT-qPCR)

Total RNA in peritoneal cells was extracted using a TRIzol reagent (Life Technologies) and immediately reverse transcribed into cDNA using the PrimeScript^™^ RT reagent kit (Takara, Kusatsu, SG, Japan). The cDNA was amplified in qPCR reactions containing TB Green Premix Ex Taq (Takara) with 0.4 μM primers on a Bio-Rad CFX96 system. The thermal cycler conditions were as follows: 5 min denaturation at 95 °C, followed by 40 cycles of 30 s at 95 °C, 30 s at 55 °C, and 30 s at 72 °C. The primers used for qPCR are shown in Additional file 1: Table S1. Relative mRNA expression was calculated by comparative quantification cycle (Cq) normalized to GAPDH using the 2^−ΔΔCq^ method.

### Arginase activity assay

Arginase activity from peritoneal cells was tested by a commercial Arginase Activity Colorimetric Assay Kit (BioVision, Milpitas, CA, USA) according to the manufacturer’s specifications. Briefly, 2 × 10^6^ cells were lysed on ice with 200 µl ice cold arginase assay buffer. After centrifugation at 10,000×*g* for 5 min, the supernatant was collected and centrifuged at 15,000×*g* for 2 min using a 10 kDa spin column to remove urea from the samples. The filtrate was discarded and the sample (retentate) was brought to its original volume with arginase assay buffer, then intermediately quantified. The unit of arginase activity was denoted as U/ml (one unit means the amount of enzyme that will generate 1.0 µmol of H_2_O_2_ per min at 37 °C).

### T cell isolation and stimulation

Spleens were isolated from control BALB/c mice under aseptic conditions and prepared for cellular suspensions through a 70 μm cell strainer (BD Biosciences, San Jose, CA, USA). Single-cell suspensions were resuspended in an erythrocyte-lysing buffer (BD Biosciences) and washed. T cells were enriched by a Pan T Cell Isolation Kit (Miltenyi Biotec) according to the manufacturer’s specifications. The purity (CD3*ε*^+^) was confirmed at > 90% by flow cytometry. Normal T cells were stimulated with 3 μg/ml anti-CD3*ε* plus 500 ng/ml anti-CD28 antibodies (BD Biosciences) and cultured in RPMI 1640 (Life Technologies) without l-arginine for 24 h. Media was supplemented with 4% fetal bovine serum (Gibco, Carlsbad, CA, USA), 100 U/ml penicillin and 100 μg/ml streptomycin.

### Co-culture in transwells

Peritoneal cells from *E. granulosus*-infected and homochronous control mice (9 months post-infection) were respectively cultured in the bottom chamber of a twelve-well transwell system containing 0.4 μm pores (Corning, New York, USA). RPMI culture medium was the same as described above, but contained 300 μM l-arginine (Sigma-Aldrich, Saint Louis, MO, USA). In some experiments, an excess of l-arginine (2 mM), the arginase inhibitor BEC (90 μM, BioVision), the NOS inhibitor l-NMMA (500 μM, BioVision) and hydrogen peroxide (H_2_O_2_) scavenger catalase (200 U/ml, Sigma-Aldrich) were respectively added in the cultures at 0 h. After 24 h, stimulated T cells in the absence of l-arginine were transferred to the upper chamber of transwells. The re-expression of CD3*ζ* in CD4^+^ and CD8^+^ T cells was tested after 48 h by flow cytometry.

### Flow cytometry analysis

Single-cell suspensions were prepared from the upper chamber of transwells and spleens of *E. granulosus*-infected and homochronous control mice at different time points. To assess the differential expression of CD3*ζ* in CD4^+^ and CD8^+^ T cells, surface staining was performed using Brilliant Violet 421 labeled anti-CD3*ε*, APC labeled anti-CD4 and PE labeled anti-CD8 (BD Biosciences). After cells were fixed and permeabilized by a Cytofix/Cytoperm^™^ Fixation/Permeabilization Kit (BD Biosciences), intracellular staining was performed with AF488 labeled anti-CD3*ζ* and isotypic control (Santa Cruz Biotechnology, Santa Cruz, CA, USA).

In addition, to assess the differential expression of ARG-1 from multiple peritoneal myeloid cells *in vivo* (3, 6, 9 and 12 months post-*E. granulosus* infection), surface staining was performed using Brilliant Violet 421 labeled anti-CD11b, APC labeled anti-Gr-1, FITC labeled anti-Ly-6G, FITC labeled anti-F4/80, PE-Cy7 labeled anti-Ly-6C and PE-Cy7 labeled anti-CD11c (BD Biosciences). Intracellular staining was performed with PE labeled anti-arginase-1 and isotypic control (Life Technologies). The percentages of SSC^high^CD11b^+^F4/80^+^ cells in total peritoneal leukocytes, SSC^low^CD11b^+^F4/80^+^, CD11b^+^CD11c^+^, CD11b^+^Gr-1^+^Ly-6C^+^Ly-6G^−^, CD11b^+^Gr-1^+^Ly-6C^−^Ly-6G^+^, CD11b^+^Gr-1^+^ and CD11b^+^Ly-6G^+^ cells in SSC-low leukocytes, together with the percentage of ARG-1 expression in corresponding cells were determined.

Fluorescence acquisition was determined using the LSRFortessa X-20 flow cytometer (BD Biosciences) and data analysis was performed using FlowJo software (BD Biosciences). Results were expressed as mean fluorescence intensity or percentage.

### Measurement of circulating free arginine, ornithine, citrulline, urea and NO

A 200 μl serum sample was thoroughly mixed with a 200 μl mixture of 0.1 mol/l HCl and 10% trichloracetic acid (1:2). The solution was centrifuged at 12,000× *rpm* for 25 min, then the supernatant was collected and standing at 4 °C for 30 min. After a second centrifugation (12,000× *rpm* for 25 min), the supernatant was collected and the presence of arginine, ornithine, citrulline and urea was determined using an ion-exchange AA analyzer (l-8900, High speed amino-acid Auto-Analyzer; Hitachi, Tokyo, Japan).

The production of NO in serum was measured using the Nitric Oxide Fluorometric Assay Kit (BioVision) according to the manufacturer’s instructions. The total concentration of nitrite is used as a quantitative measure of NO production that is completely converted to nitrite.

### Statistical analysis

Comparisons of arginase expression and activity, CD3*ζ* expression, concentrations of arginine, ornithine, citrulline, urea and NO were performed *via* a one-way or two-way ANOVA, or a two-tailed Student’s t-test using the GraphPad Prism statistical programs (GraphPad Software, San Diego, CA, USA). Data were represented as the mean ± standard deviation (SD). A *P*-value < 0.05 was considered statistically significant.

## Results

### Genotype identification

Comparative analysis using nucleotide sequences deposited in the GenBank database, the Eg-PSC used for infection in the present study was identified as genotype G1.

### ARG-1 was upregulated in multiple peritoneal cells post-infection *in vivo*

**Fig. 1:**
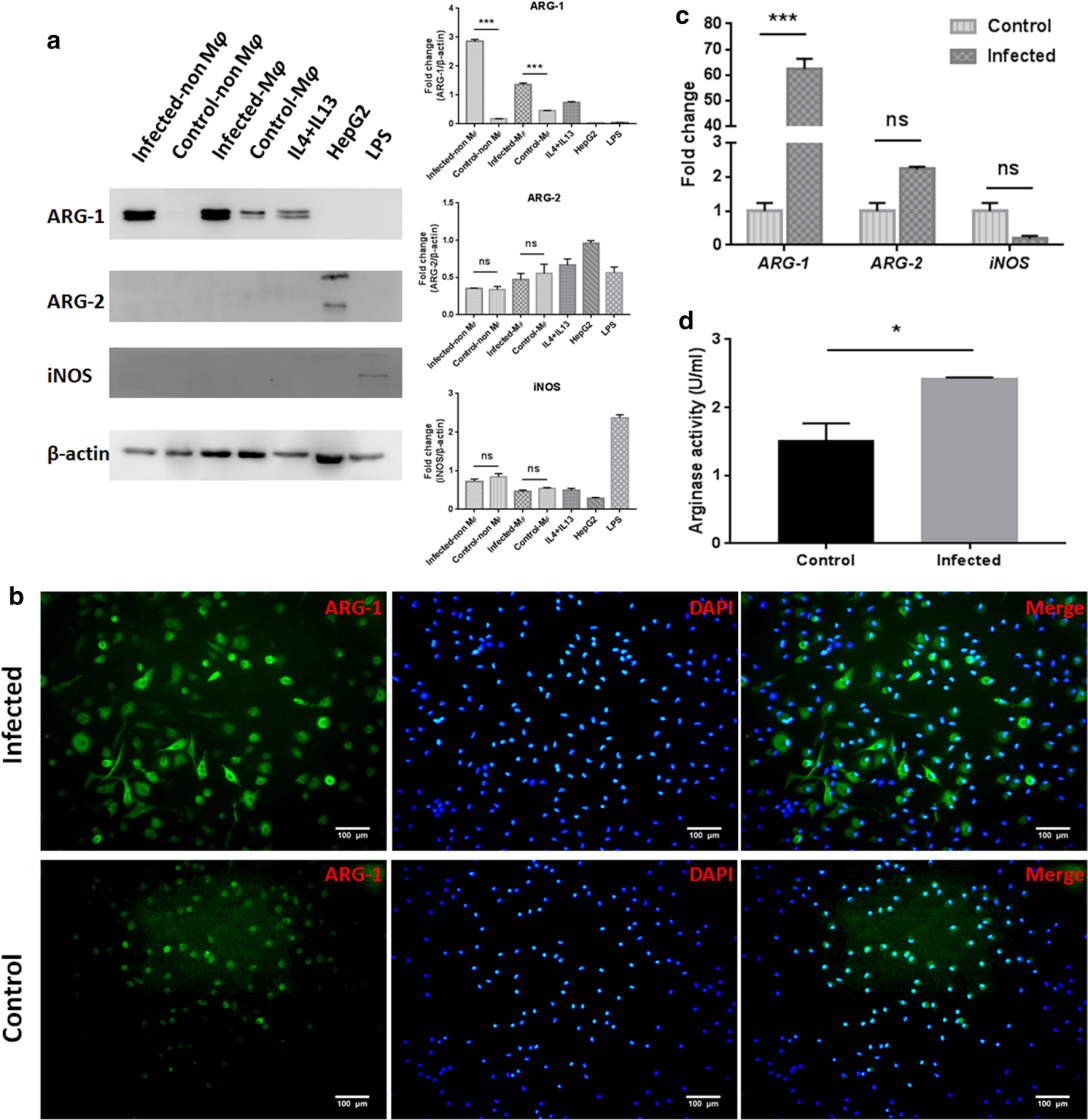
Presence and activity of arginase in peritoneal cells (9 months post-infection). **a** Immunoblot of ARG-1, ARG-2 and iNOS from the lysates of macrophages and non-macrophage cells from infected and control mice (macrophages were magnetically separated from peritoneal cells and the remaining cells were labeled non-macrophage cells). The IL4 and IL13 stimulated peritoneal cells, HepG2 cells and LPS stimulated peritoneal cells served as positive controls. Graphical representations of band intensities are shown in the right panel. Protein expression was normalized to β-actin. Differences were analyzed by a one-way ANOVA. **b** Immunofluorescent assay of ARG-1 in whole peritoneal cells. DAPI was used to visualize nuclei. *Scale-bars*: 100 µm. **c** The levels of *ARG-1*, *ARG-2* and *iNOS* mRNA expression in peritoneal cells were analyzed by RT-qPCR. Relative mRNA expression was normalized to GAPDH. The difference was analyzed by a two-way ANOVA. **d** 2 × 10^6^ cell lysates in 200 µl arginase assay buffer were used to determine arginase activity. The unit of arginase activity is denoted as U/ml (one unit means the amount of enzyme that will generate 1.0 µmol of H_2_O_2_ per min at 37 °C). The difference was analyzed by a Student’s t-test. The data are the result of a representative experiment out of three independent experiments. *P < 0.05, **P < 0.01, ***P < 0.001. *Abbreviations*: ns, not significant

To confirm the differential expression of arginase in peritoneal cells *in vivo*, a western blot analysis, immunofluorescent assay and RT-qPCR were used to determine the expression level in mice infected with Eg-PSC and homochronous control mice (9 months post-infection). Considering that macrophages make up the majority of peritoneal cells, macrophages and non-macrophage cells were detected, respectively, for arginase and iNOS by western blot analysis. Enhanced ARG-1 expression (protein level) was observed in macrophages and non-macrophage cells from infected mice compared with that in the control group (*F*_(6, 14)_ = 2272, *P* < 0.001), whereas the expression of ARG-2 and iNOS were unchanged (Fig. 1a). In wild-type control mice, ARG-1 was mainly present in macrophages. The immunofluorescent assay for whole peritoneal cells also confirmed increased ARG-1 expression in infected mice compared with that in control mice (Fig. 1b), while the ARG-2 expression remained unchanged (Additional file 2: Figure S1). RT-qPCR results exhibited enhanced *ARG-1* (*F*_(2, 6)_ = 683.6, *P* < 0.001), unchanged *ARG-2* and *iNOS* mRNA levels in infected mice compared with those in control mice (Fig. 1c). Meanwhile, higher arginase activity was found in peritoneal cells from infected mice than that in the control group (*t*_(4)_ = 3.367, *P* = 0.019) (Fig. 1d).

**Fig. 2:**
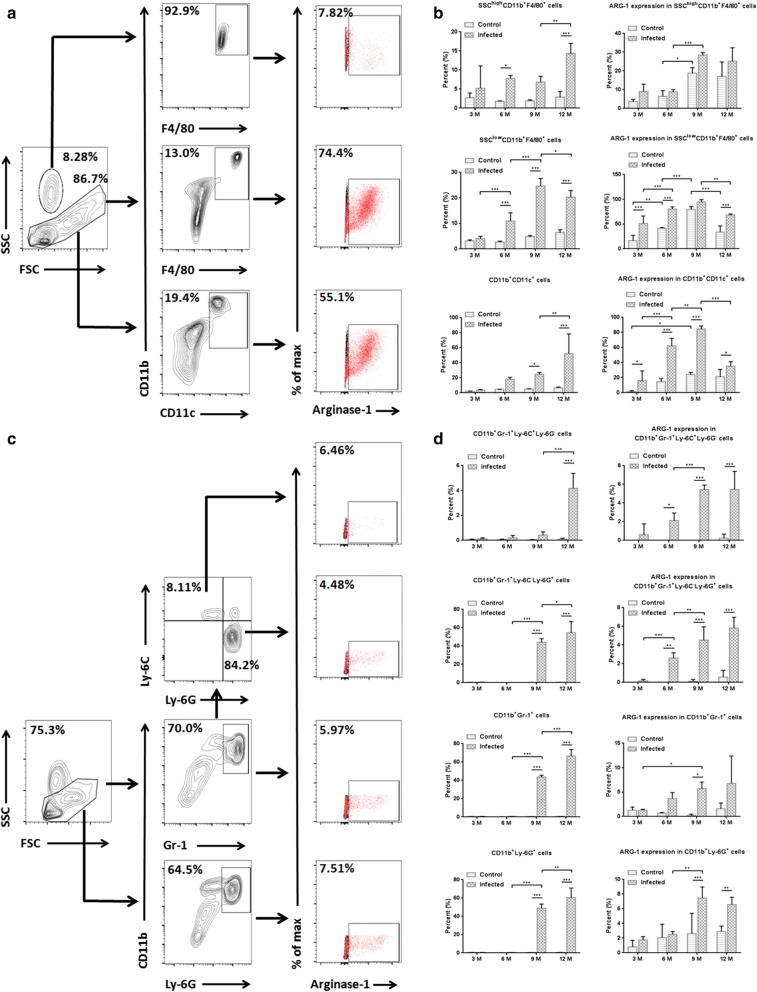
ARG-1 is produced in multiple peritoneal myeloid cells at different time points post-infection. **a** Representative flow cytometry analysis of ARG-1 expression in SSC^high^CD11b^+^F4/80^+^, SSC^low^CD11b^+^F4/80^+^ and CD11b^+^CD11c^+^ cells from one infected mouse at 6 months post-infection. **b** The percentages of SSC^high^CD11b^+^F4/80^+^ cells in total peritoneal leukocytes, SSC^low^CD11b^+^F4/80^+^ and CD11b^+^CD11c^+^ cells in SSC-low leukocytes, together with the percentages of ARG-1 expression in corresponding cells were determined by flow cytometry at 3, 6, 9 and 12 months post-infection. The differences were analyzed by a two-way ANOVA. **c** Representative flow cytometry analysis of ARG-1 expression in CD11b^+^Gr-1^+^Ly-6C^+^Ly-6G^−^, CD11b^+^Gr-1^+^Ly-6C^−^Ly-6G^+^, CD11b^+^Gr-1^+^ and CD11b^+^Ly-6G^+^ cells from one infected mouse at 12 months post-infection. **d** The percentages of CD11b^+^Gr-1^+^Ly-6C^+^Ly-6G^−n^, CD11b^+^Gr-1^+^Ly-6C^−^Ly-6G^+^, CD11b^+^Gr-1^+^ and CD11b^+^Ly-6G^+^ cells in SSC-low leukocytes, and the percentages of ARG-1 expression in the corresponding cells were determined by flow cytometry at 3, 6, 9 and 12 months post-infection. The differences were analyzed by a two-way ANOVA. The percentages of ARG-1 expression (red) were confirmed on the basis of isotypic controls (black) in **a** and **c**. The data are the result of one representative experiment out of three independent experiments. *P < 0.05, **P < 0.01, ***P < 0.001

To further characterize the population producing ARG-1 in peritoneal cells, flow cytometry analysis was performed for multiple myeloid cells at different time points post-infection (3, 6, 9 and 12 months). Enhanced expression of ARG-1 was found in SSC^low^CD11b^+^F4/80^+^, CD11b^+^CD11c^+^, CD11b^+^Gr-1^+^Ly-6C^+^Ly-6G^−^, CD11b^+^Gr-1^+^Ly-6C^−^Ly-6G^+^, CD11b^+^Gr-1^+^ and CD11b^+^Ly-6G^+^ cells in infected mice compared with that in control mice (Fig. 2b, d). Compared to that at 3 months post-infection, increased expression of ARG-1 was observed in SSC^low^CD11b^+^F4/80^+^, CD11b^+^CD11c^+^ and CD11b^+^Gr-1^+^Ly-6C^−^Ly-6G^+^ cells from infected mice at 6 months (*F*_(3, 12)_ = 3.963, *P* < 0.001; *F*_(3, 12)_ = 26.29, *P* < 0.001; and *F*_(3, 12)_ = 16.46, *P* < 0.001, respectively). Compared to those at 6 months post-infection, SSC^high^CD11b^+^F4/80^+^, CD11b^+^CD11c^+^, CD11b^+^Gr-1^+^Ly-6C^+^Ly-6G^−^, CD11b^+^Gr-1^+^Ly-6C^−^Ly-6G^+^ and CD11b^+^Ly-6G^+^ cells from infected mice exhibited an enhanced percentage of ARG-1 expression at 9 months (*F*_(3, 12)_ = 1.209, *P* < 0.001; *F*_(3, 12)_ = 26.29, *P* = 0.002; *F*_(3, 12)_ = 17.48, *P* < 0.001; *F*_(3, 12)_ = 16.46, *P* = 0.007; and *F*_(3, 12)_ = 12.44, *P* = 0.003, respectively). A decreased level of ARG-1 expression was found in SSC^low^CD11b^+^F4/80^+^ and CD11b^+^CD11c^+^ cells from infected mice at 12 months compared to that at 9 months post-infection (*F*_(3, 12)_ = 3.963, *P* = 0.002 and *F*_(3, 12)_ = 26.29, *P* < 0.001, respectively). In addition, in infected mice, the percentages of SSC^high^CD11b^+^F4/80^+^ cells in total peritoneal leukocytes, SSC^low^CD11b^+^F4/80^+^, CD11b^+^CD11c^+^, CD11b^+^Gr-1^+^Ly-6C^+^Ly-6G^−^, CD11b^+^Gr-1^+^Ly-6C^−^Ly-6G^+^, CD11b^+^Gr-1^+^ and CD11b^+^Ly-6G^+^ cells in SSC-low leukocytes, exhibited a rising trend along with the extension of infection time, except for a slight fluctuation in SSC^low^CD11b^+^F4/80^+^ cells at 12 months post-infection. Taken together, these data verified that ARG-1 is upregulated in multiple peritoneal cells after Eg-PSC infection and exhibits a gradually rising trend prior to 9 months.

### CD3*ζ* was downregulated in CD4^+^ and CD8^+^ T cells after 9 months post-infection *in vivo*

**Fig. 3:**
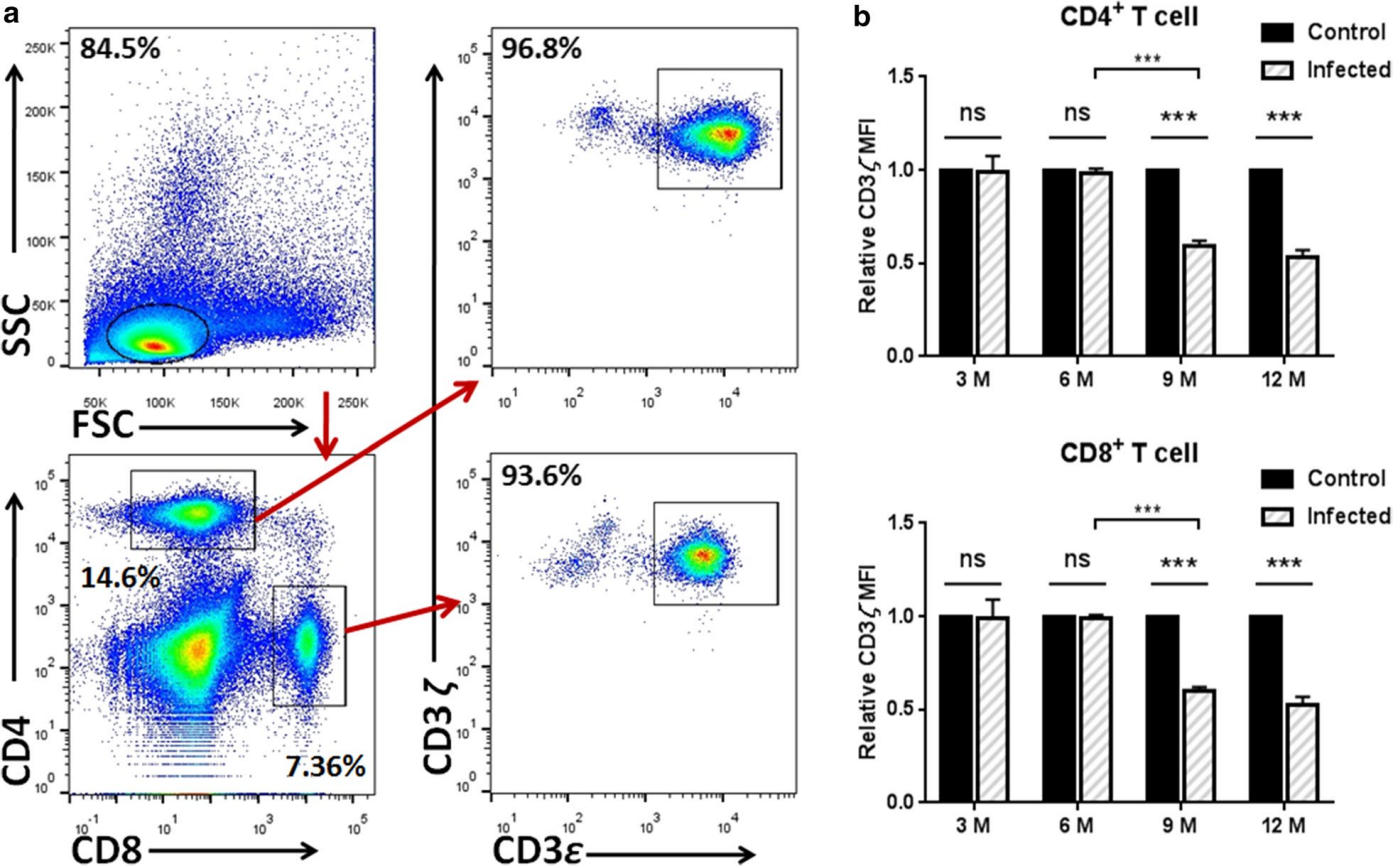
Dynamic studies of CD3*ζ* expression in CD4^+^ and CD8^+^ T cells *in vivo*. **a** Representative flow cytometry analysis of CD3*ζ* expression in CD4^+^ (CD3*ε*^+^CD4^+^) and CD8^+^ (CD3*ε*^+^CD8^+^) T cells among spleen leukocytes from one infected mouse at 6 months post-infection. **b** The relative MFI of CD3*ζ* expression in CD4^+^ and CD8^+^ T cells was quantified by flow cytometry at different time points post-infection (3, 6, 9 and 12 months). The differences were analyzed by a two-way ANOVA. The data are the result of one representative experiment out of three independent experiments. *P < 0.05, **P < 0.01, ***P < 0.001. *Abbreviations*: ns, not significant

After 3, 6, 9 and 12 months post-infection, mice (including the homochronous control mice) were respectively sacrificed to determine differential expression of CD3*ζ* in CD4^+^ and CD8^+^ T cells from spleens. Kinetic studies showed that the relative expression of CD3*ζ* remained unchanged in CD4^+^ and CD8^+^ T cells during the first 6 months after Eg-PSC infection, and the decline occurred at 9 and 12 months in the infected group compared to the control group (*F*_(3, 12)_ = 94.88, *P* < 0.001 and *F*_(3, 12)_ = 75.54, *P* < 0.001, respectively) (Fig. 3).

### Arginase blocked CD3*ζ* re-expression in activated T cells *in vitro*

**Fig. 4:**
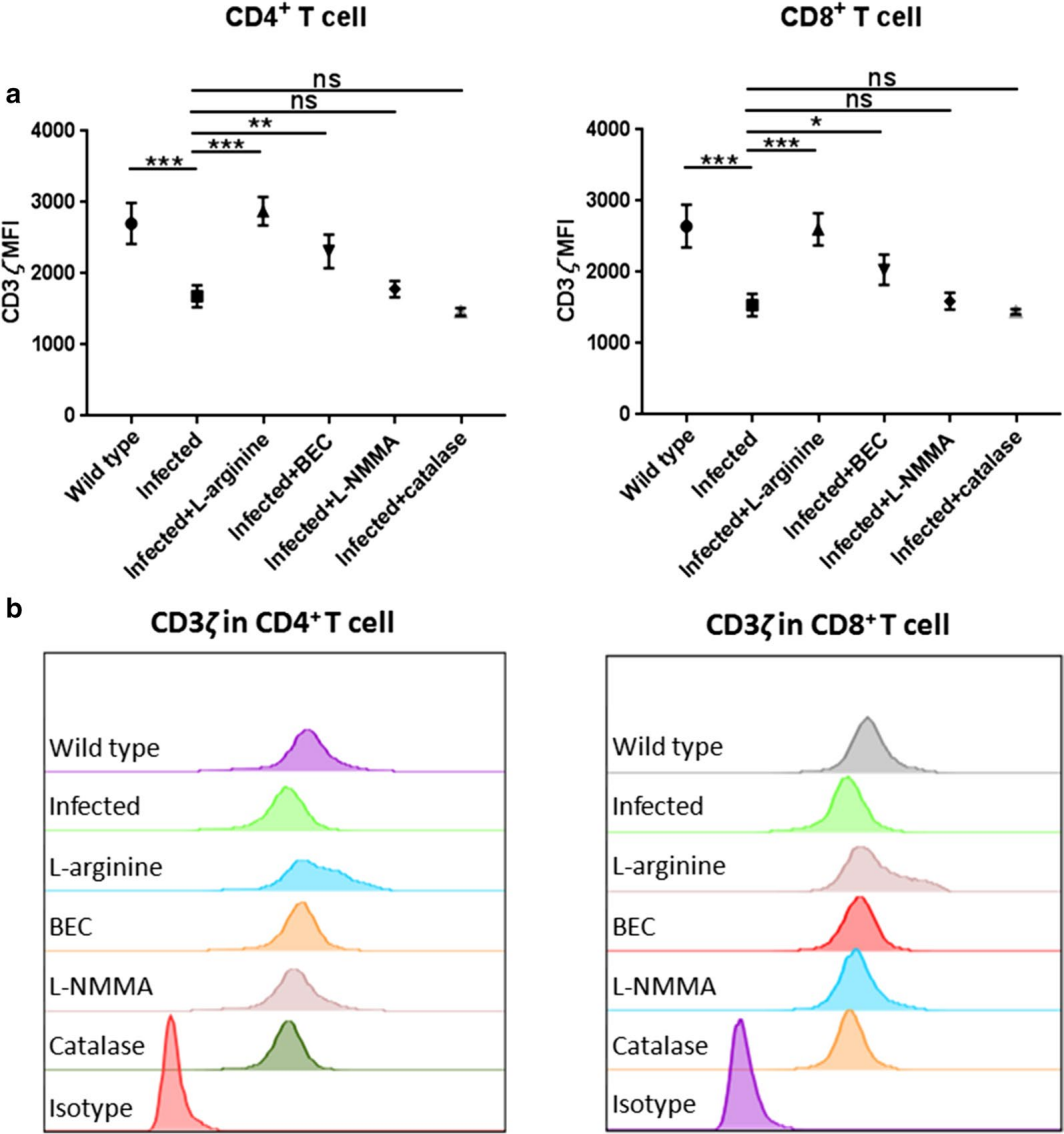
Arginase produced by peritoneal cells from infected mice prevents the re-expression of the CD3*ζ* chain. Isolated murine pan T cells were stimulated with 3 μg/ml anti-CD3*ε* plus 500 ng/ml anti-CD28 antibodies in l-arginine-free RPMI 1640 for 24 h, and peritoneal cells from infected/control mice were cultured in RPMI 1640 containing 300 μM l-arginine separately for 24 h. Stimulated T cells were then added to the upper chamber of a co-culture transwell system containing the peritoneal cells in the bottom chamber for an additional 48 h of culture. In some experiments, the l-arginine (2 mM), BEC (90 μM), l-NMMA (500 μM) and catalase (200 U/ml) were respectively added into the cultures at 0 h. **a** CD3*ζ* re-expression in CD4^+^ and CD8^+^ T cells was tested by flow cytometry. The differences were analyzed by a one-way ANOVA. **b** Representative histograms of CD3*ζ* re-expression in activated CD4^+^ and CD8^+^ T cells co-cultured with peritoneal cells for 48 h. The data are the result of one representative experiment out of three independent experiments. *P < 0.05, **P < 0.01, ***P < 0.001. *Abbreviations*: ns, not significant

In conventional RPMI 1640 media containing l-arginine, T cells stimulated with anti-CD3*ε*^+^ and anti-CD28^+^ antibodies undergo a cycle of internalization and re-expression of CD3*ζ*. However, this will be blocked in the absence of l-arginine, preventing the assembly of new T cell receptors [20]. To confirm the role of arginase involved in immunosuppression due to Eg-PSC infection, freshly isolated peritoneal cells from infected or control mice were co-cultured with activated T cells in a transwell system. When compared to the control group, an ability of CD4^+^ and CD8^+^ T cells to re-express CD3*ζ* was blocked in the Eg-PSC-infected group (*F*_(5, 12)_ = 27.52, *P* < 0.001 and *F*_(5, 12)_ = 23.16, *P* < 0.001, respectively). Nevertheless, this effect was prevented by the excess exogenous l-arginine and the arginase inhibitor BEC (for CD4^+^ T cells: *F*_(5, 12)_ = 27.52, *P* < 0.001 and *P* = 0.007; for CD8^+^ T cells: *F*_(5, 12)_ = 23.16, *P* < 0.001 and *P* = 0.035), but not by the addition of the NOS inhibitor l-NMMA nor the hydrogen peroxide scavenger catalase, demonstrating that the depletion of l-arginine by arginase from *E. granulosus*-associated peritoneal cells impaired the re-expression of T cell receptor CD3*ζ* chain (Fig. 4).

### l-arginine metabolism was skewed toward a preferential urea/ornithine production

**Fig. 5:**
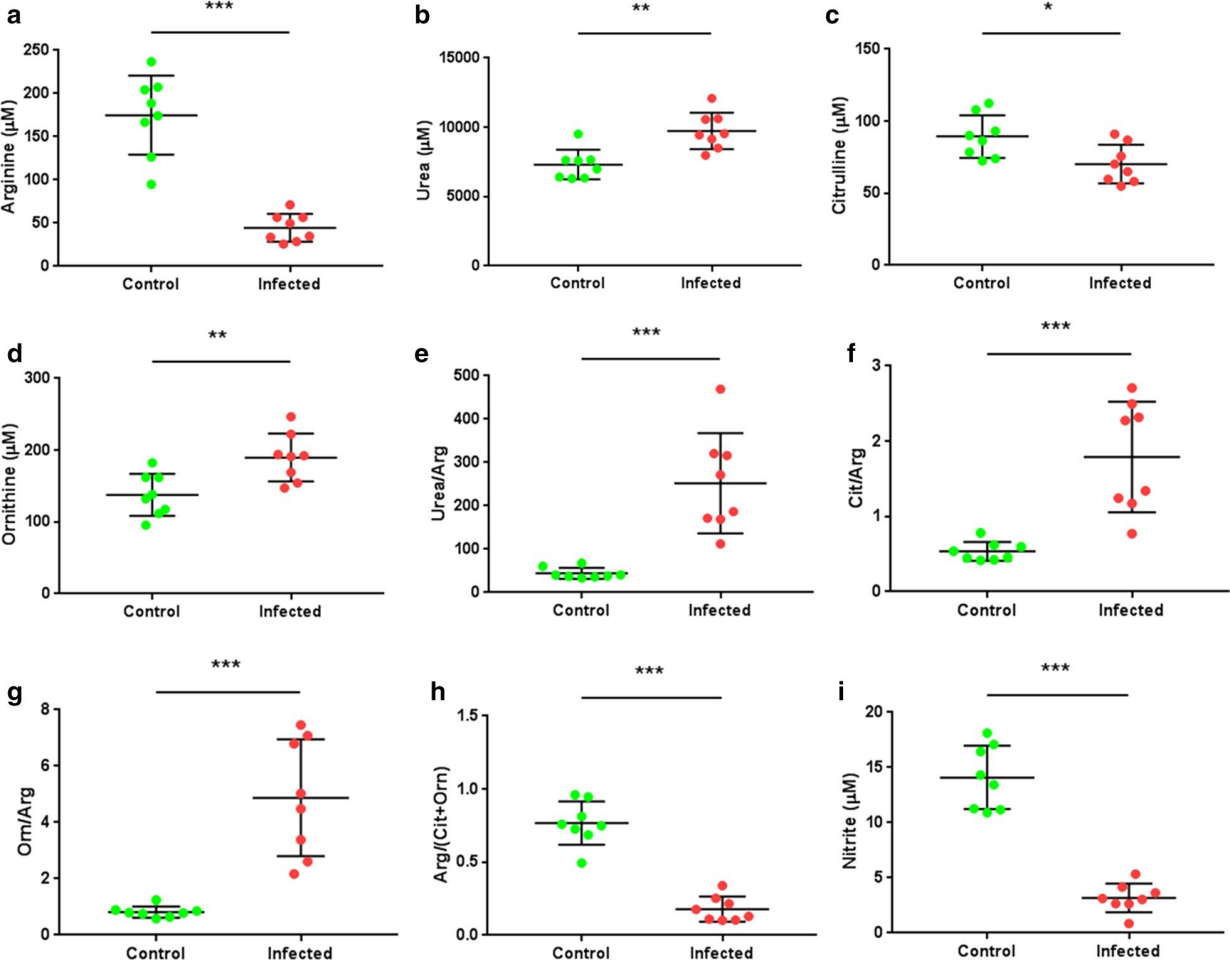
Concentrations of arginine-related metabolites in serum from mice (9 months post-infection). The concentration of arginine (**a**), urea (**b**), citrulline (**c**) and ornithine (**d**) was quantified by an ion-exchange AA analyzer. **e** The concentration ratio between urea and arginine (Arg). **f** The ratio between citrulline (Cit) and arginine. **g** The ratio between ornithine (Orn) and arginine. **h** The ratio of arginine/(citrulline + ornithine). **i** The total concentration of nitrite, used as a quantitative measure of NO production. The differences were analyzed by a Student’s t-test. *P < 0.05, **P < 0.01, ***P < 0.001

To validate the systematic changes of l-arginine and its related metabolites in serum, the concentrations of amino acids, urea and NO were assessed in a quantitative profiling between Eg-PSC-infected mice (*n* = 8) and controls (*n* = 8) 9 months post-infection. The concentration of l-arginine was significantly decreased in the infected group (less than 50 μM) compared with that in the control group (*t*_(14)_ = 7.581, *P* < 0.001). Meanwhile, a decreased concentration of citrulline and NO, and an increased concentration of urea and ornithine were observed in the infected group (*t*_(14)_ = 2.711, *P* = 0.017; *t*_(14)_ = 9.781, *P* < 0.001; *t*_(14)_ = 4.059, *P* = 0.001; and *t*_(14)_ = 3.302, *P* = 0.005, respectively), consistent with elevated urea/arginine ratio, citrulline/arginine ratio, ornithine/arginine ratio and decreased arginine/(citrulline + ornithine) ratio (Fig. 5). These results verified that l-arginine metabolism in Eg-PSC-infected mice is skewed toward a preferential urea/ornithine production, in agreement with elevated arginase expression and activity *in vivo*.

## Discussion

One of the hallmarks during parasite infection is persistence. Previous studies have revealed that the hydatid cysts survival in hosts is prolonged by complex evasion mechanisms, such as physical and immunological protection by the laminated layer [21], blocking the penetration of IgG by the germinal layer [22], modulation by antigen-presentation cells, antigen B and cytokines [23]. The present study supports that ARG-1 is also involved in immunosuppression in mice infected with *E. granulosus*.

The impact of ARG-1 and ARG-2 on pathogen control, host survival and immune response has been studied in several parasitoses. For example, during murine *Toxoplasma gondii* infection, ARG-1 in macrophages thwarted effective immunity, impaired parasite control, and elimination of *ARG-1* favored host survival [24]. On the contrary, in infections with the *Trichuris muris* or *Leishmania major*, mice lacking ARG-1 or ARG-2 showed unaltered cytokine responses, parasite burden and NO production [11, 25]. Here, our data showed elevated expression (protein and mRNA levels) and activity of ARG-1 in peritoneal cells from mice with *E. granulosus* infection. Nevertheless, ARG-2 and iNOS were unchanged. Similarly, only ARG-1 was found in abnormal spleens and livers from *E. granulosus-*infected mice, as described in previous studies [17, 18]. Moreover, compared with the control mice, the fold change of ARG-1 expression in non-macrophage cells from infected mice increased more than that in macrophages, due to high level of ARG-1 expression in macrophages from control mice as shown in Figs. 1a, 2b (F4/80^+^). Flow cytometry analysis detected enhanced production of ARG-1 in SSC^low^CD11b^+^F4/80^+^, CD11b^+^CD11c^+^, CD11b^+^Gr-1^+^Ly-6C^+^Ly-6G^−^, CD11b^+^Gr-1^+^Ly-6C^−^Ly-6G^+^, CD11b^+^Gr-1^+^ and CD11b^+^Ly-6G^+^ cells, but no significant change in SSC^high^CD11b^+^F4/80^+^ cells from infected mice compared with that in control mice. Elevated arginase expression was consistently found in macrophages, dendritic cells, granulocytes and immature myeloid cells (myeloid suppressor cells) under other models or stimuli, depleting l-arginine [26–28].

The active and inactive stages of hydatid disease are dominated by different immune responses. Th1 response benefits the host, facilitating the death and clearance of Eg-PSC in mice, whereas Th2 response benefits the parasite, allowing it to survive in the host [29]. In patients with cystic echinococcosis, antigen B has been shown to impair the Th1 response by upregulating Th2 cytokines IL-4 and IL-13 which could stimulate ARG-1 production *in vitro* [30, 31]. For Eg-PSC-infected mice, the Th1-type cytokine profile was predominant at the early phase (3–4 weeks post-infection), in the 4th week this shifted to a Th2-type cytokine profile [32]. In the chronic phase, we found that the expression of ARG-1 exhibits a rising trend in multiple peritoneal myeloid cells along with the extension of infection time, except for fluctuations in SSC^low^CD11b^+^F4/80^+^ and CD11b^+^CD11c^+^ cells at 12 months post-infection.

The suppressive activity of arginase has mainly been correlated with l-arginine depletion, which leads to downregulation of T cell receptor CD3*ζ* chain expression in many diseases [33–35]. However, some tumors with high ASS1 activity are able to produce l-arginine from citrulline and are not affected by the high level of arginase [12]. In the present study, we showed that the relative expression of CD3*ζ* chain in CD4^+^ and CD8^+^ T cells decreased at 9 and 12 months after *E. granulosus* infection compared with that in the control group, whereas no difference was detected at 3 and 6 months. Notably, at the later period (9 months post-infection), reduced CD3*ζ* was associated with elevated ARG-1 expression (Fig. 1a) and a significantly decreased l-arginine concentration in serum (Fig. 5a). At the first 6 months, the degree of ARG-1 metabolism might be not enough to impair T cell function, which needs further experimental verification. Rodriguez et al. [36] showed that the concentration of l-arginine below 40 μM caused a rapid decrease of CD3*ζ* chain *in vitro*, consistent with rodents and patients undergoing trauma or liver transplantation [37, 38]. *In vitro*, ARG-1, but not iNOS or H_2_O_2_, produced by *E. granulosus*-associated peritoneal cells prevented the re-expression of CD3*ζ* chain in CD4^+^ and CD8^+^ T cells, regenerated by the supplement of l-arginine or BEC. The hydrogen peroxide scavenger catalase was included in some experiments because H_2_O_2_ released by activated macrophages and neutrophils has been shown to impair CD3*ζ* expression in previously published reports [39, 40]. A similar effect has been observed in a tumor model, where the addition of exogenous l-arginine or arginase inhibitors could recover the CD3*ζ* loss and T cell proliferation was reestablished [36]. Although the availability of l-arginine influenced CD3*ζ* expression and T cell function, and initial reports suggested that the low expression of CD3*ζ* was associated with impaired T cell function [41, 42], the causal relationship between them has not been completely defined and requires further research. Studies have shown that T cells also exhibited early activation status marked by CD25, CD69, CD122, CD132 and increased IL-2 production, when cultured in l-arginine-free media [4]. Meanwhile, when such T cells were stimulated by phorbol myristate acetate, which bypassed T cell receptor signaling, they also failed to proliferate, meaning some other mechanisms may present in causing T cell dysfunction triggered by the absence of l-arginine [6].

l-arginine is mainly converted by arginase and NOS to l-ornithine and urea, NO and l-citrulline, respectively. The defensive role of NO in the host against *E. granulosus* larvae was previously reported [43]. *In vitro*, the laminated layer was found to reduce NO production [44] and increase arginase expression [43]. At the later stage of Eg-PSC infection, in the present study, we found the arginine pathway in serum exhibited more arginase metabolites and less NO/citrulline in infected mice than those in control mice, indicating that the antagonism of both enzymes is present in *E. granulosus-*infected mice. The same result appeared in severe fever with thrombocytopenia syndrome (SFTS) cases, where the global arginine bioavailability ratio (GABR) was used as a good prognostic marker for fatal prediction in early SFTS virus infection [45]. Taken together, these results corroborate that l-arginine metabolism post-infection is skewed toward a preferential production of ornithine/urea instead of NO/citrulline, which is beneficial to parasite survival.

## Conclusions

This study provide important data showing increased expression of ARG-1 in peritoneal cells after Eg-PSC infection. In addition, along with the extension of infection time, the expression of ARG-1 showed a rising trend in multiple myeloid cells, whereas the expression of the CD3*ζ* chain showed a decreasing trend. Moreover, the ARG-1 produced by *E. granulosus*-associated peritoneal cells not only inhibited the re-expression of CD3*ζ* by metabolism, but antagonized iNOS. Understanding the underlying mechanism of arginase in immune evasion of *E. granulosus* infection will pave the way for targeted treatment.

## Supplementary information

**Additional file 1: Table S1.** Primers used in qPCR detection.

**Additional file 2: Figure S1.** Immunofluorescent assay of ARG-2 in whole peritoneal cells (9 months post-infection). DAPI was used to visualize nuclei.

## Data Availability

Data supporting the conclusions of this article are included within the article and its additional files.

## Abbreviations

ARG: Arginase
iNOS: Inducible nitric oxide synthase
Eg-PSC: *Echinococcus granulosus* protoscoleces
PBS: Phosphate-buffered saline
BEC: S-(2-boronoethyl)-l-cysteine
l-NMMA: l-monomethyl-l-arginine
DAPI: 4’,6-diamidino-2-phenylindole, dihydrochloride
cDNA: Complementary deoxyribonucleic acid
GAPDH: glyceraldehyde-3-phos-phate dehydrogenase
M-MDSCs: Monocytic myeloid-derived suppressor cells
NO: Nitric oxide
ASS: Arginine-succinate synthetase
IL: Interleukin
LPS: Lipopolysaccharide
