# Supplementary figures and images for: Arginase promotes immune evasion of Echinococcus granulosus in mice
Source: Parasit Vectors. 2020 Feb 6;13:49. doi: 10.1186/s13071-020-3919-4 (PMC7006169; doi:10.1186/s13071-020-3919-4)

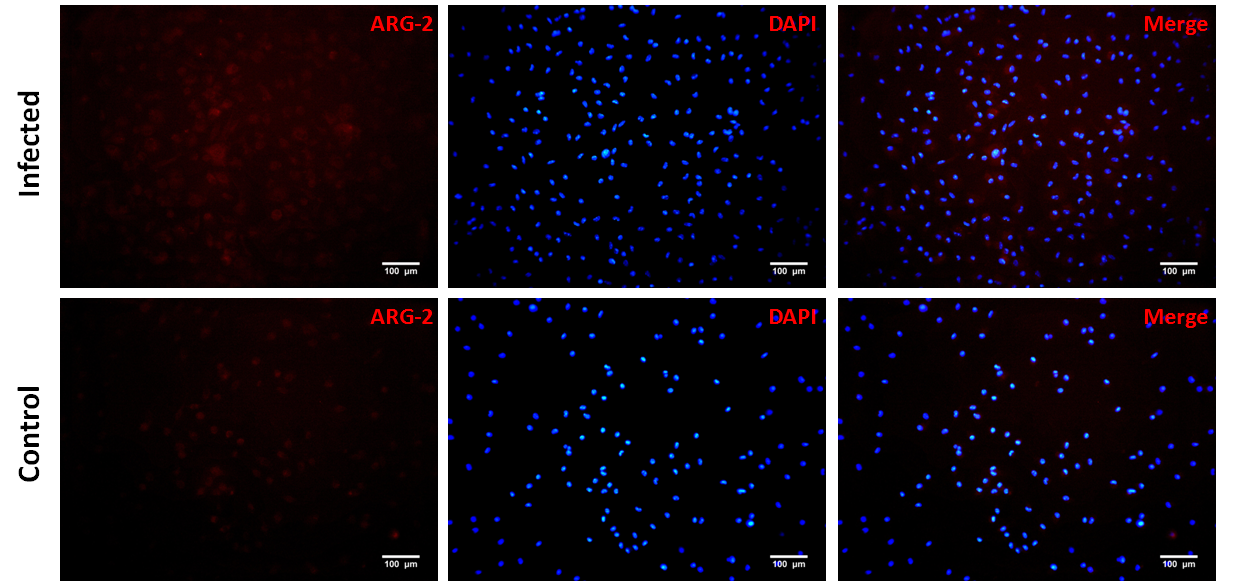

Supplement: Supplementary file 2 — Additional file 2: Figure S1. Immunofluorescent assay of ARG-2 in whole peritoneal cells (9 months post-infection). DAPI was used to visualize nuclei. [file 13071_2020_3919_MOESM2_ESM.tif]
